# Endothelial IL-8 induced by porcine circovirus type 2 affects dendritic cell maturation and antigen-presenting function

**DOI:** 10.1186/s12985-019-1256-z

**Published:** 2019-12-12

**Authors:** Shiyu Liu, Qiuming Li, Jinzeng Qiao, Jianfang Wang, Defeng Cui, Kewei Gu, Shuanghai Zhou, Huanrong Li

**Affiliations:** 1 College of Animal Science and Technology, Beijing University of Agriculture, No. 7 Beinong Road, Changping District, Beijing, 102206 China; 2 Beijing Key Laboratory of TCVM, Beijing University of Agriculture, No. 7 Beinong Road, Changping District, Beijing, 102206 China

**Keywords:** Porcine circovirus type 2, Monocyte-derived dendritic cells, Endothelial IL-8, Maturation, Antigen presentation

## Abstract

**Background:**

Porcine circovirus (PCV) disease caused by PCV type 2 (PCV2) is mainly attributed to immunosuppression and immune damage. PCV2 can infect vascular endothelial cells and induce high expression of endothelial IL-8. Dendritic cells (DCs), as professional antigen-presenting cells, can not only present antigens but also activate naïve T-cells, causing an immune response.

**Methods:**

To demonstrate whether endothelial IL-8 is the main factor inhibiting the maturation and related functions of dendritic cells during PCV2 infection, monocyte-derived DCs (MoDCs) and porcine iliac artery endothelial cells (PIECs) processed by different methods were co-cultured in two ways. Flow cytometry, molecular probe labeling, fluorescence quantitative PCR, and the MTS assay were used to detect the changes in related functions and molecules of MoDCs.

**Results:**

Compared to those in the PIEC-DC group, the endothelial IL-8 upregulation co-culture group showed significantly lower double-positive rates for CD80/86 and MHC-II of MoDCs and significantly increased endocytosis of MoDCs. Meanwhile, the adhesion rate and average fluorescence intensity of MoDCs were significantly downregulated in migration and adhesion experiments. Furthermore, the MHC-I and LAMP7 mRNA levels in MoDCs and the proliferation of MoDC-stimulated T-cells were markedly reduced. However, the changes in MoDCs of the endothelial IL-8 downregulation co-culture group were the opposite.

**Conclusions:**

PCV2-induced endothelial IL-8 reduces the adhesion and migration ability of MoDCs, resulting in a decreased maturation rate of MoDCs, and further inhibits antigen presentation by DCs. These results may explain the immunosuppressive mechanism of PCV2 from the perspective of the interaction between endothelial cells and DCs in vitro.

## Background

Porcine circovirus (PCV) disease caused by PCV type 2 (PCV2) leads to major economic losses in the pig industry worldwide, mainly due to the immunosuppression and immunopathological damage in infected piglets [1, 2]. Its clinical manifestations include post-weaning multi-systemic wasting syndrome (PMWS) [3], porcine dermatitis and nephropathy syndrome (PDNS) [4], reproductive disorders, and porcine respiratory disease complex (PRDC) [5]. In recent years, the study of the immunosuppression mechanism caused by PCV2 has become the focus of effective control and prevention of PCV2. Currently, vaccine inoculation is an effective means of preventing and controlling PCV2 infection, but vaccines can only alleviate lymphoid tissue damage, viremia, and PCV2 replication in vivo and not block the spread of PCV2 and clear the virus in pigs [6]. To further enhance the effect of the vaccine, some adjuvants are usually added to the vaccine. Studies have confirmed that cytokines have an obvious immune adjuvant effect and are good immunopotentiators. The current research on cytokine adjuvants mainly focuses on interferons and interleukins [7, 8]. Therefore, it is very important to study the role of cytokines in pathogenesis for better development of vaccines and prevention of diseases.

DCs can efficiently take up, process, and present antigens; activate naïve T-cells; and induce immune responses [9]. These biological functions are closely related to in vivo migration [10]. At first, DCs reach the peripheral tissues from the hematopoietic organs and then from the latter reach the T-cell area of the lymph nodes. In the initial process, DCs are immature and have a high capacity to capture antigens. Thereafter, DCs gradually mature and have a high ability to process and present antigens and activate naïve T-cells [11]. DCs can deliver antigen peptides not only to CD4^+^ Th cells through binding of the major histocompatibility complex (MHC)-II to antigen peptides, but also to CD8^+^ Tc cells by binding of MHC-I class molecules to antigen peptides [12], which play an important role in the body’s antiviral immunity. However, some viruses, such as Herpes virus [13], influenza virus [14], and human immunodeficiency virus [15], can evade or delay immune responses by disrupting DC function [16, 17].

Endothelial IL-8 produced by endothelial cells and fibroblasts is quite different from leukocytic IL-8. Studies have shown that while endothelial IL-8 mediates weak chemotaxis, it can induce apoptosis and inhibit the maturation of DCs in humans [18, 19]. PCV2 can cause arterial endotheliitis in infected pigs and can be detected in endothelial cells [20]. Porcine iliac artery endothelial cells (PIECs) can inhibit the maturation of monocyte-derived DCs (MoDCs), and PCV2-infected PIECs can further inhibit MoDC maturation, while PCV2-infected PIECs can highly express endothelial IL-8 [21]. Therefore, this study used two different co-culture methods to detect the changes in MoDCs by flow cytometry, molecular probe labeling, fluorescence quantitative PCR, and the MTS assay. It aimed to elucidate whether endothelial IL-8 was the main molecule involved in the inhibition of DC maturation after PIECs were infected with PCV2. This will improve the immunopathogenesis of PCV2.

## Materials and methods

### Virus and animals

The PCV2-SD/2008 strain (GenBank accession number: GQ174519) used in this study was isolated and identified by Laboratory of Animal Infectious Diseases at Hebei Agricultural University according to the reported study [22]. The viral stock was prepared in PK-15 cells with a titer of 10^5.5^ TCID_50_/ml. Six healthy, 21-day-old, Large White-Dutch Landrace crossbred weaning piglets were used and their treatment methods at the end of the experiment were consistent with those used previously [21], that is, the piglets were euthanized by intravenously injecting an excess of sodium pentobarbital (70 ± 80 mg/kg, Sinopharm Chemical Reagent Beijing Co., Beijing, China).

### Cells

The culture of PIECs and isolation of peripheral blood mononuclear cells (PBMCs) were performed in accordance with the methods introduced in the literature [21]. PIECs were obtained from the Cell Resource Center of Shanghai Institutes for Biological Sciences (Shanghai, China, Catalog number: GN105) and maintained in RPMI 1640 (GIBCO, Grand land, NY, USA) supplemented with 10% heat-inactivated fetal bovine serum (FBS) (Sigma, Missouri, USA) and 200 U of penicillin-streptomycin/ml at 37 °C in a humidified 5% CO_2_ incubator (Thermo, New York, USA).

PBMCs were isolated with EDTA-anticoagulant peripheral blood from the front cavity vein of each piglet by density centrifugation over Ficoll-Paque (1.077 g/L) at 400×g for 20 min, and then labeled with fluorescein isothiocyanate (FITC)-CD14 antibody (Bio-Rad, California, USA). CD14^+^ monocytes were fractionated by magnetic separation using an MS column (Miltenyi, Bergisch-Gladbach, Germany) and their purity was more than 90%. The isolated monocytes were stained by trypan blue exclusion, and the concentration was 1 × 10^7^ cells/ml for inducing MoDCs. Relying on previous studies [23] and our pre-experimental results, MoDC induction medium (10% FBS RPMI1640 medium containing 5 ng/ml porcine granulocyte-macrophage colony-stimulating factor [GM-CSF] (R&D, Minnesota, USA) and 3.75 ng/ml recombinant porcine IL-4 (R&D, Minnesota, USA) was used to induce MoDCs in a humidified 5% CO_2_ atmosphere at 37 °C.

### Preparation of different PIECs

#### PCV2-PIECs

PIECs were seeded (1 × 10^5^ cells/well) into a 24-well plate. After the cells reaching approximately 50–70% confluence were infected with PCV2-SD/2008 at a multiplicity of infection of 0.2 for 1 h, the infection medium was washed away and the cells were incubated in a humidified 5% CO_2_ atmosphere at 37 °C with RPMI 1640 containing 10% FBS (complete medium).

#### IL-8^over^-PIECs

According to the literature [24], a plasmid containing the porcine endothelial cell-derived IL-8 gene fragment (Lenti-OE-IL-8) was transfected into 293 T cells (human renal epithelial cell line purchased from Nanjing Keke Biotechnology Co., Ltd.). After 3 days, the supernatant was harvested and concentrated to obtain the Lenti-OE-IL-8 virus. PIECs were infected by the Lenti-OE-IL-8 virus and passaged three times. Significantly increased IL-8 gene and protein expression was observed with RT-PCR and enzyme-linked immunosorbent assay (ELISA), meaning IL-8 overexpression in PIECs was successfully established and the cells were named IL-8^over^-PIECs.

#### IL-8^si^-PIECs

PIECs were seeded on 24-well plates in 10% FBS RPMI 1640 without penicillin/streptomycin, and were incubated in a humidified 5% CO_2_ atmosphere at 37 °C overnight. When the cells reached 60% confluence, they were treated with 80 μM small interfering RNA (siRNA) per well in RPMI 1640. Equimolar amounts of siRNA were incubated with Lipofectamine 2000 Transfection Reagent (Madison, WI, United States) according to the manufacturer’s instructions. The transfection efficiency was detected by fluorescence microscopy, reverse transcription polymerase chain reaction (RT-PCR), and ELISA. When the IL-8 expression inhibition rate of PIEC reached 60% or more, the IL-8 protein level significantly downregulated, and IL-8^si^-PIECs could be used for subsequent experiments. IL-8 siRNA (sense: 5′-CGAUGCCAGUGCAUAAAUATT-3′, antisense: 5′-UAUUUAUGCACUGGCAUCGTT-3′) and negative (control) siRNA (sense: 5′-UUCUUCGAACGUGUCACGUTT-3′, antisense: 5′-ACGUGACACGUUCGGAGAATT-3′) were designed and synthesized by Shanghai Genepharma (Shanghai, China).

#### Ab-IL-8-PIECs

During co-culture with MoDCs, IL-8 neutralizing antibody (5 μg/ml, Abcam, Cambridge, USA) was added to the MoDC induction medium.

### Co-culture of PIECs and MoDCs

The 0.1-μm pore size Transwell membranes (Millipore, Massachusetts, USA) were suspended in 24-well plates (Corning, New York, USA). Monocytes were seeded into the upper reservoir of the Transwell membrane with the MoDC induction medium. Two co-cultivation methods (after-induction co-culture and with-induction co-culture) were used to confirm the relationship between endothelial cell-derived IL-8 and MoDC-related functions. The former involved adding the sorted monocytes into the upper chamber of Transwell, after which these cells were induced in the MoDC induction medium, and half of the medium was changed every other day for 5 days. The cells were then co-cultured with different pre-treated PIECs in a humidified 5% CO_2_ atmosphere at 37 °C for 48 h. In the latter approach, the isolated monocytes were added to the upper chamber of Transwell and directly co-cultured with the different pre-treated PIECs in a 24-well plate for 5 days. The induced medium was the same as the former. The ratio of the upper and lower cells was 1:10. MoDCs from different co-culture groups were collected and counted by the trypan blue exclusion method for further study. Meanwhile, DCs from the induced monocytes and the PCV2-infected DCs were also included.

According to the different PIECs, the experiment was divided into seven groups: PIEC-DC, PCV2-PIEC-DC, IL-8^over^-PIEC-DC, Ab-IL-8-PIEC-DC, IL-8^si^-PIEC-DC, DC alone, and PCV2-DCs. Among them, PCV2-PIEC-DCs and IL-8^over^-PIEC-DCs belonged to the endothelial IL-8 upregulation groups, and Ab-IL-8-PIEC-DCs and IL-8^si^-PIEC-DCs belonged to the endothelial IL-8 downregulation groups.

### Detection of MoDC phenotype molecules

CD1a, SWC3a, CD80/86, and MHC-II on the surface of MoDCs were determined using flow cytometry (Beckman-Coulter, California, USA). Mouse anti-porcine FITC-CD1a antibody and FITC-anti-Monocyte/Granulocyte antibody (Abcam, Cambridge, UK) were used to detect DCs. Mouse anti-porcine FITC-SLA-DR antibody (AbD Serotec, Kidlington, UK) and the R-PE-CD152 (CTLA-4)-muIg (Ancell, California, USA) were used for the detection of DC maturation. Isotype control antibodies mouse IgG1-FITC, mouse IgG2a-FITC (Abcam, Cambridge, UK), mouse IgG2b-FITC (AbD Serotec, Kidlington, UK), and mouse IgG1-PE (Ancell, California, USA) were used for background control.

### Endocytosis of MoDCs

After the MoDCs were collected and counted, 150 μl of each sample cell suspension and 150 μl of FITC-Dextran (Sigma, Missouri, USA) (molecular weight, 40 KD; concentration, 1 mg/ml) were mixed and incubated in a humidified atmosphere of 5% CO_2_ at 37 °C for 1 h. After centrifugation at 1000 r/min for 10 min, the cells were resuspended with 200 μl of RPMI 1640 medium and the percentage of FITC^+^ cells in each group was detected by flow cytometry to analyze the endocytosis of MoDCs.

### Adhesion of MoDCs

According to a previous study [25], the monolayer of PIECs was incubated with the molecular probe Green 5-chloromethyl fluorescein diacetate (CMFDA; LP0S6ate, Abcam, Cambridge, UK) at 37 °C for 45 min. The different derived MoDCs were incubated with dihydroethidium (DHE, Beyotime, Shanghai, China) 37 °C for 45 min, respectively. After the labeled MoDCs (200 μl) were co-incubated with the labeled PIEC monolayer for 4 h, the supernatant was discarded and the unattached MoDCs were gently washed and fixed with 4% paraformaldehyde for 20 min. The adhesion was visualized under a fluorescence microscope (adhesion ratio = the number of MoDCs/the number of PIECs).

### Migration of MoDCs

The different groups of MoDCs were incubated with DHE at 37 °C for 45 min, centrifuged at 2000 rpm for 10 min, resuspended with serum-free RPMI1640 medium, and were adjusted to 5 × 10^5^/ml. Labeled MoDCs (100 μl) were added to the pre-paved PIECs of the 24-well Transwell (5 μm) upper chamber and serum-free RPMI 1640 containing 200 ng/ml MCP-1 (Peprotech, New Jersey, USA) was added to the lower chamber. After incubation at 37 °C for 8 h, the relative fluorescence intensity of the cells in the lower chamber was determined as the relative migration quantity of the cells by using a multifunctional automated quantitative plate reader (PHERAstar; BMG Labtech, Offenburg, Germany).

### Extraction of total RNA and real-time fluorescence quantitative RT-PCR (FQ RT-PCR)

The total RNA of MoDCs in the different groups above was respectively extracted according to the RNA extraction kit instructions (Aidlab, Beijing, China), and was reverse-transcribed to synthesize cDNA according to the instructions of the reverse transcription kit (Cwbio, Beijing, China). Referring to a previously reported method [26], the molecules (LMP7, UBP, MHC-I, calreticulin, and β-actin; primers are shown in Table 1) were detected by real-time FQ RT-PCR.

**Table 1:** Swine-specific primer sequences used for quantitative SYBR ROX-1 real-time PCR

Gene source/accession no.	Accession number	Primer sequence (5′-3′)	Annealing temperature (°C)	Products (bp)
LMP7	AF059493	For: AGTGATTGTGGCGGTGGATT	56	328
		Rev: CCGAGTCCCATTTTCATCCA		
UBP	AF134195	For: GTGAGAACTGTGGCAGGAAGACC	58	347
		Rev: TTCCCAGGACACCCAACAGA		
MHC-I	AY135587	For: GAGGGGCAGGAGTATTGGGATAG	58	309
		Rev: CCTCAATTGCTCCGCCACAT		
Calreticulin	GQ984146	For: ATGACTGCTAGGTGTTTAAAATTA	56	226
		Rev: GGATCTCTGGCAGGTCAAGT		
β-actin	U 07786	For: TCATCACCATCGGCAACT	59	547
		Rev: TTCCTGATGTCCACGTCGC		

Note: β-actin was used as the internal control

bp: base pairs; for: forward primer; rev: reverse primer

### Mixed lymphocyte reaction

2 × 10^6^ T-cells/ml enriched from PBMCs of allogeneic animals by using a nylon wool column (Nylon Fiber Column T [L-Type], Wako, Japan) were mixed with 2 × 10^5^ DCs/ml in 96-well plates at the ratio of 10:1. T-cells without stimulator cells were used as an untreated control. The cultures were incubated in growth medium at 37 °C in an atmosphere of 5% CO_2_ for 3 days.

Proliferation was measured using the Cell Titer 96 AQueous One Solution Assay (Promega, Wisconsin, USA). Twenty microliters of 3-(4, 5-dimethylthiazol-2-yl)-5-(3-carboxymetho-xyphenyl)-2-(4-sulfophenyl)-2H-tetrazolium inner salt (MTS) was added to each well and incubated for another 4 h. The optical density (OD_490_nm) was recorded using a microplate reader (Bio-Rad, California, USA). Raw data were calculated as the stimulation index (SI) = (OD_treated_ – OD_blank_)/(OD_untreated_ – OD_blank_).

### Statistical analysis

The data are presented as mean ± standard deviation (SD) values of three independent experiments. For the analysis of mRNA expression levels, we used the absolute quantitative method. Raw data were normalized against the values obtained for β-actin and the transcription levels were expressed as the ratio of molecules to β-actin mRNA expression, and the fold-changes of the other groups over the DC alone group were seen as the relative values of each molecule to compare mRNA levels between groups. A one-way analysis of variance was used to determine whether the differences between groups were statistically significant. *P* values < 0.05 were considered significant.

## Results

### Endothelial IL-8 induced by PCV2 inhibited the maturation of MoDCs

**Fig. 1:**
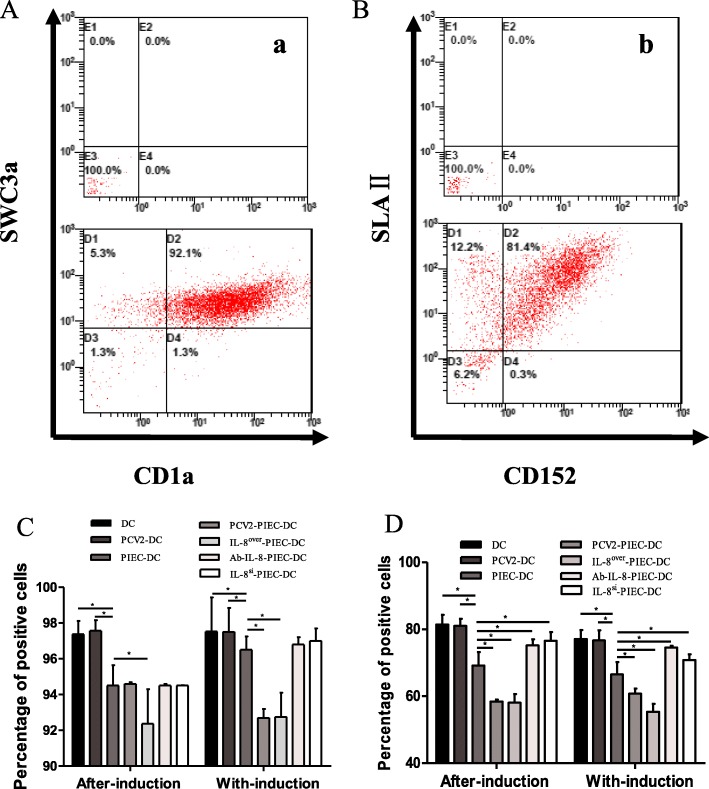
Dot plots and percentage of dendritic cells expressing surface markers. Flow cytometric analysis was conducted to detect double-positive staining for surface markers (A: CD1a and SWC3a; B: MHC-II and CD80/86; a and b: background control); flow cytometry was used to determine the percentage of monocyte-derived dendritic cells (MoDCs) staining positive for MHC-II (C) or CD80/86 (D). Data are presented as the mean and standard deviation (error bars) for each group. Error bars represent the standard deviation. * indicates *P* < 0.05. The data are shown as the mean ± standard deviation of three independent experiments

As seen in Fig. 1A, more than 90% of MoDCs were positive for both CD1a and SWC3a, which indicated MoDCs had been induced successfully. In both co-culture modes, the expression rates of MHC-II and CD80/86 in all co-culture groups were significantly lower than those in the single culture groups. In the after-induction co-culture, the expression rates of MHC-II in the IL-8^over^-PIEC-DCs were significantly lower than those in the PIEC-DCs, while in the with-induction co-culture, the expression rates of MHC-II in the endothelial IL-8 upregulation groups were significantly lower than those in the PIEC-DCs. The expression rates of CD80/86 were different from those of MHC-II (Fig. 1C). The expression rates in the endothelial IL-8 upregulation groups were significantly lower than those in the PIEC-DCs, while the expression rates in the endothelial IL-8 downregulation groups were significantly higher than those in the PIEC-DCs (Fig. 1D). The significant decrease of MHC-II and CD80/86 expression in the endothelial IL-8 upregulation groups suggested that endothelial IL-8 induced by PCV2 could inhibit the maturation of MoDCs.

### Endothelial IL-8 induced by PCV2 enhanced MoDC endocytosis

**Fig. 2:**
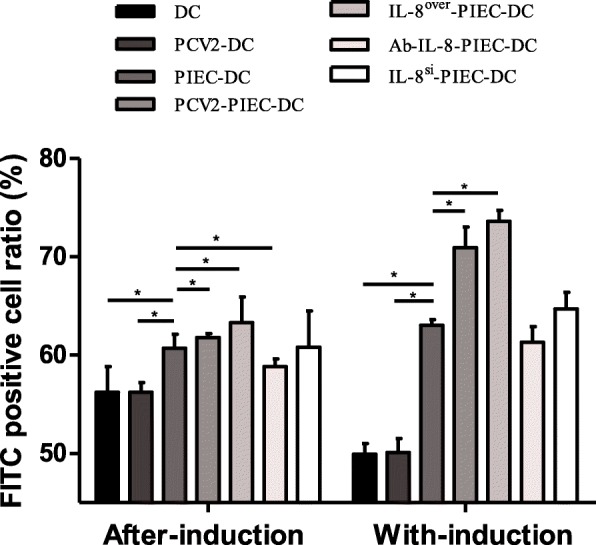
Changes in MoDC endocytosis in both co-culture modes. MoDCs were collected and incubated with FITC-dextran for 1 h, and FCM was used to detect the FITC-positive cell ratio in each group. Data are presented as the mean and standard deviation (error bars) for each group. Error bars represent the standard deviation. * indicates P < 0.05. The data are shown as the mean ± standard deviation of three independent experiments

In the two co-cultivation methods, the FITC-positive rates in the co-culture groups were significantly higher than those in the single culture groups. The FITC-positive rates in the endothelial IL-8 upregulation groups were significantly higher than that in the PIEC-DC group. On the other hand, the corresponding rates in the endothelial IL-8 downregulation groups were lower than that in the PIEC-DC group except for that in the IL-8^si^-PIEC-DCs of the with-induction co-culture groups, and the rate in the Ab-IL-8-PIEC-DCs of the after-induction co-culture groups was significantly different (Fig. 2). The results above implied that endothelial IL-8 induced by PCV2 could enhance the endocytosis of MoDCs.

### Endothelial IL-8 induced by PCV2 inhibited MoDC adhesion

**Fig. 3:**
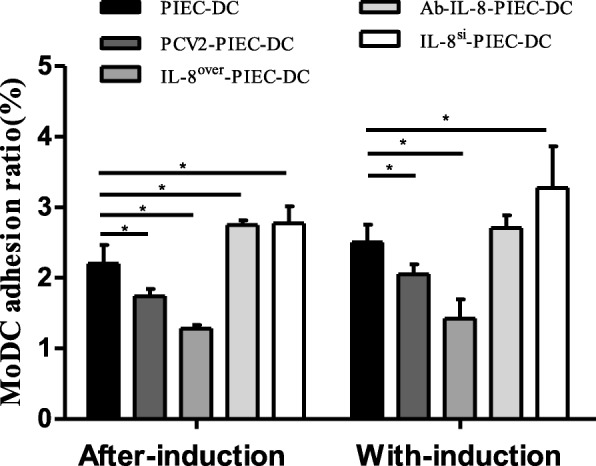
Changes in the adhesion rates of MoDCs. The adhesion rate was calculated by fluorescent microscopy using the fluorescent probe labeling method. Data are presented as the mean and standard deviation (error bars) for each group. Error bars represent the standard deviation. * indicates P < 0.05. The data are shown as the mean ± standard deviation of three independent experiments

**Fig. 4:**
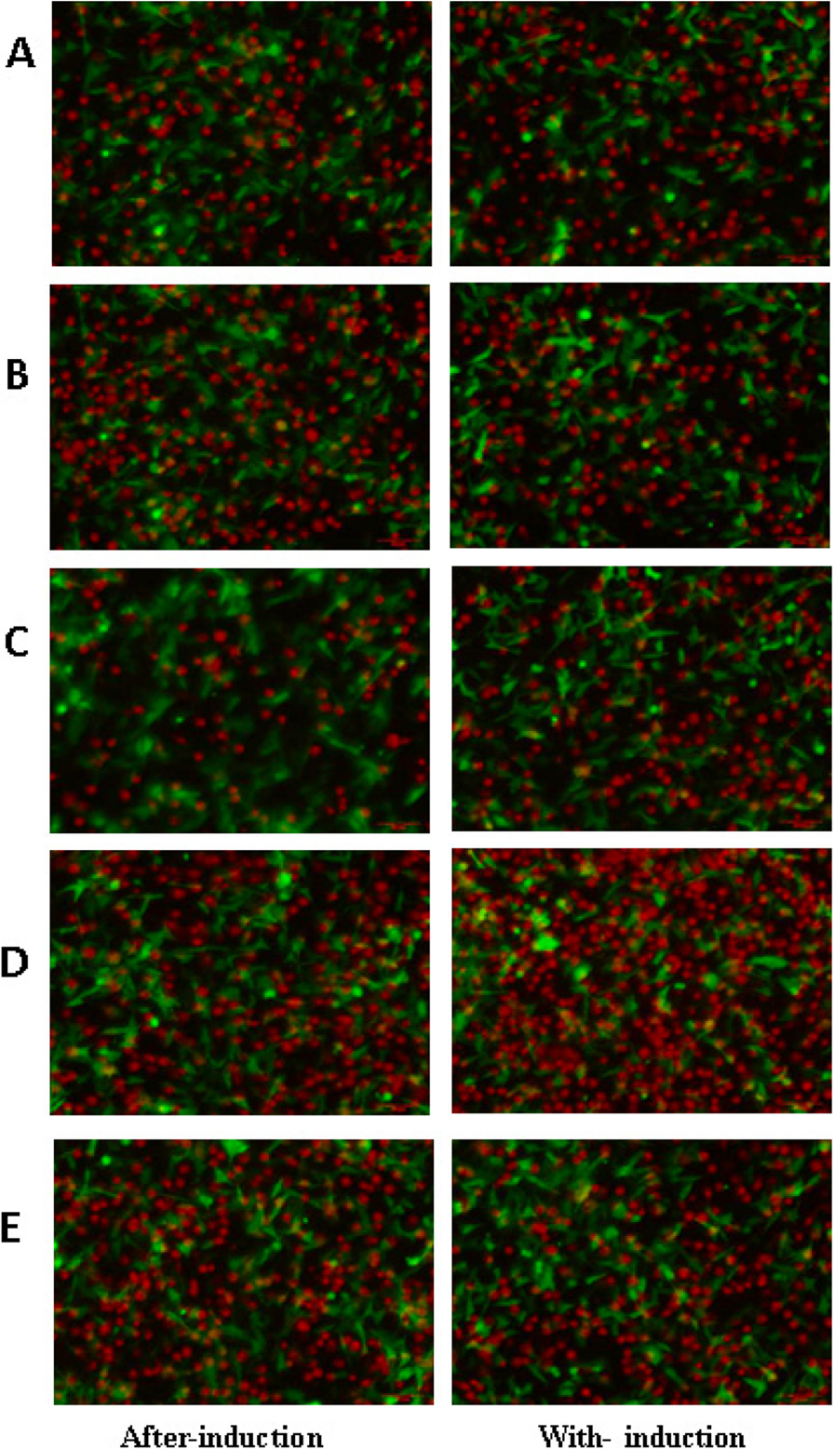
Adhesion diagram of MoDCs. Fluorescent microscopy was used to observe MoDC adhesion using the fluorescent probe labeling method. Red represents MoDCs, Green represents PIECs. A: PIEC-DC, B: PCV2-PIEC-DC, C: IL-8^over^-PIEC-DC, D: Ab-PIEC-DC, E: IL-8^si^-PIEC-DC. Scar bar = 50 μm

The molecular probe-labeled MoDCs were co-cultured with PIECs and a confocal microscope was used for examination. Figure 3 showed that the change trends in the two co-culture modes were basically the same. The adhesion rate in the PIEC-DCs was significantly higher than those in the endothelial IL-8 upregulation groups, and was significantly lower than those in the endothelial IL-8 downregulation groups except for that in the Ab-IL-8-PIEC-DC of the after-induction co-culture. The fluorescence image is shown in Fig. 4. These results demonstrated that PCV2-induced endothelial IL-8 inhibited MoDC adhesion.

### Endothelial IL-8 induced by PCV2 inhibited MoDC migration

**Fig. 5:**
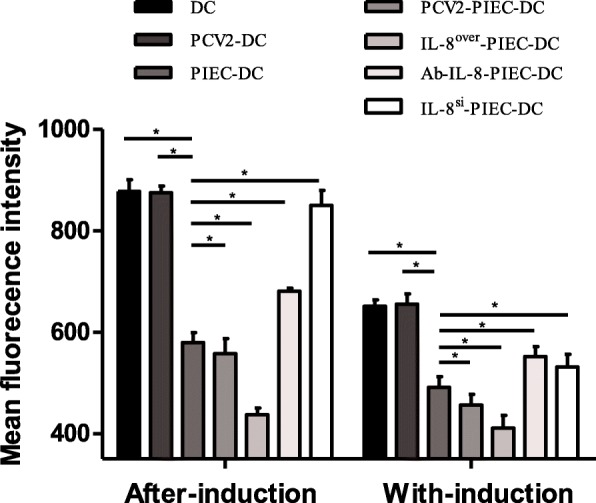
Changes in MoDC migration. PIECs were seeded into 5.0-μm Transwell (24-well) chambers and cultured for 12 h After cell fusion, fluorescently labeled MoDCs were added to the upper chamber. Transmigration in the lower chamber (supplemented with MCP-1) was stopped after 8 h. A multifunctional microplate reader was used to detect the average cell fluorescence intensity. * indicates P < 0.05. The data are shown as the mean ± standard deviation of three independent experiments

In both co-culture methods, the average fluorescence intensity of the single culture groups was significantly higher than that of the co-culture groups. The average fluorescence intensities in the endothelial IL-8 upregulation groups were significantly lower than that in the PIEC-DCs, while those in the endothelial IL-8 downregulation groups were significantly higher than that in the PIEC-DCs (Fig. 5). These results demonstrated that endothelial IL-8 inhibited the migration of MoDCs, and the higher the amount of IL-8 expression, the stronger the inhibition.

### PCV2-induced endothelial IL-8 inhibited the endogenous antigen-presenting function of MoDCs

**Fig. 6:**
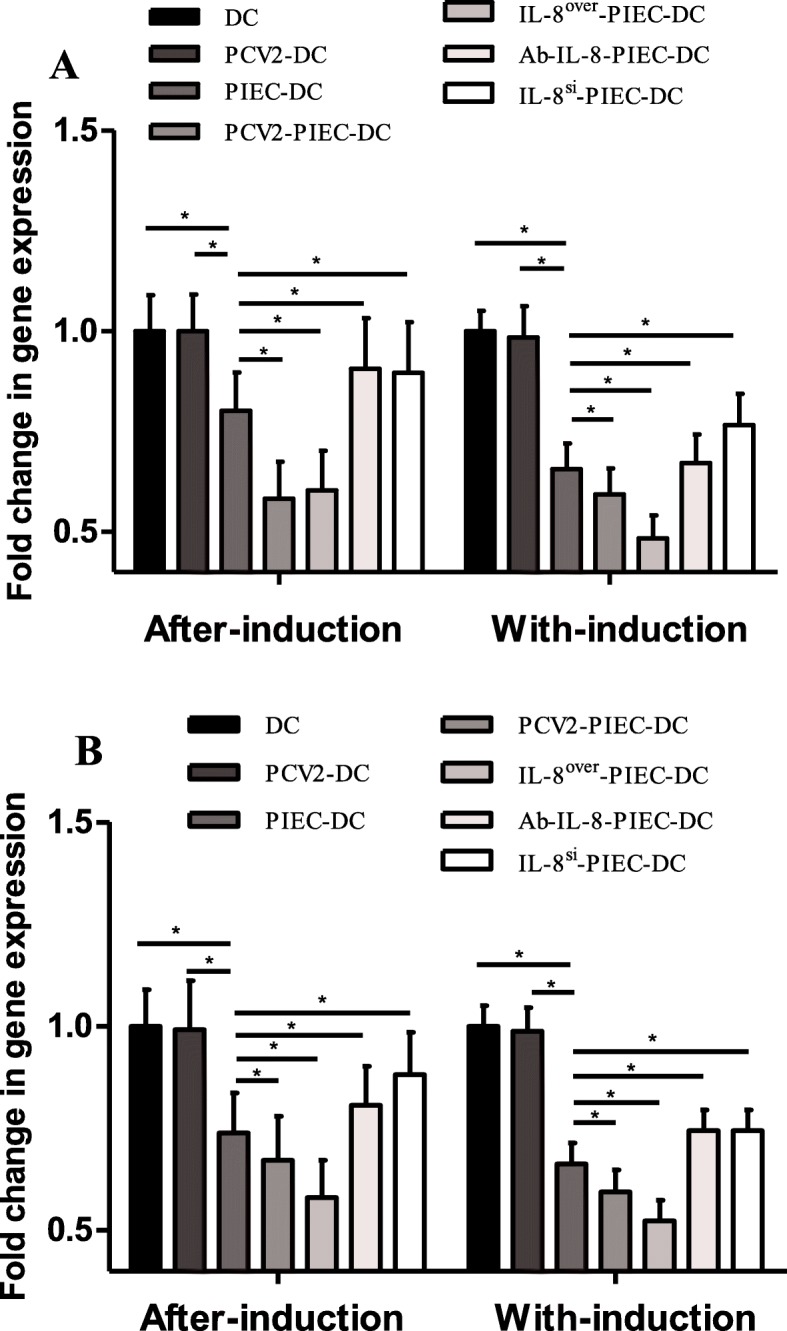
Changes in LMP7 and MHC-I mRNA expression in the collected MoDCs. mRNA expression levels of LMP7 and MHC-I were measured by quantitative real-time PCR. (A) Levels of LMP7 mRNA expression. (B) Levels of MHC-I mRNA expression. Data are expressed as the mean fold-change in gene expression in collected MoDCs (*n* = 3 per group). Error bars represent the standard deviation. * indicates P < 0.05

LMP7, MHC-I, UBP, and calreticulin are major molecules of the endogenous antigen-presentation pathway of DCs. In both co-culture methods, compared to the levels in the PIEC-DCs, the expression levels of LMP7 and MHC-I in the endothelial IL-8 upregulation groups were significantly lower (*P* < 0.05) but those in the endothelial IL-8 downregulation groups were substantially higher (P < 0.05) (Fig. 6A and B). However, there was no significant difference in the expression levels of UBP and calreticulin. These findings implied that endothelial IL-8 induced by PCV2 inhibited the endogenous MoDC antigen-presentation function.

### PCV2-induced endothelial IL-8 down-regulated the stimulatory effect of MoDCs on T lymphocytes

**Fig. 7:**
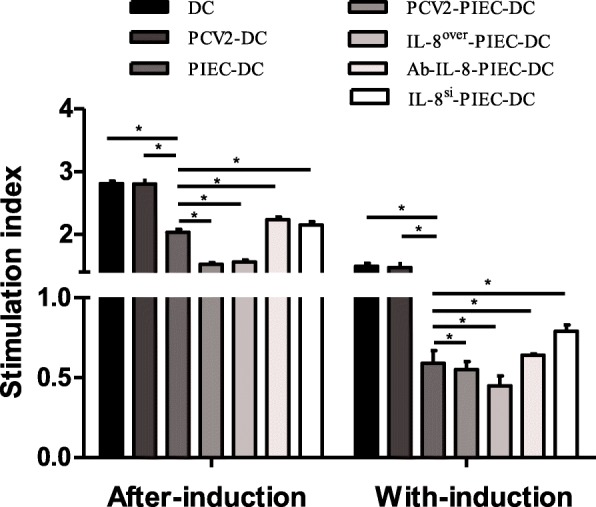
Stimulation index of T lymphocyte reaction. MTS was used to detect the stimulation index of T lymphocyte reaction. Data are presented as the mean and standard deviation (error bars) for each time point indicated. Error bars represent the standard deviation. * indicates P < 0.05. The data are shown as the mean ± standard deviation of three independent experiments

The results of T-cell proliferation test showed that the SIs of after-induction co-cultures were > 1, and the SIs of with-induction co-cultures were < 1. The SIs of the co-culture groups were significantly lower than those of the single culture groups, regardless of whether the co-culture was performed after-induction or with-induction. Moreover, the SIs of the endothelial IL-8 upregulation groups were significantly lower than that of the PIEC-DC group, while those of the endothelial IL-8 downregulation groups were significantly higher than that of the PIEC-DC group (Fig. 7). These results indicated that PIECs could inhibit the proliferation of T-cells stimulated by DCs, whereas the inhibition of PCV2-induced endothelial IL-8 was stronger.

## Discussion

Using MoDCs co-cultured with PIECs processed by different methods, we found that the PCV2-induced endothelial IL-8 could affect MoDC maturation by inhibiting their adhesion and migration ability. The immature MoDCs showed high endocytosis ability and low antigen-presentation capacity, and eventually decreased the proliferation of T lymphocytes. These results may explain the immunosuppressive mechanism of PCV2 from the perspective of the interaction between endothelial cells and DCs in vitro.

CD80/86 and MHC-II double-positivity are indicators of MoDC maturity and their weak endocytosis [16, 27]. Vincent reported that the surface molecules of PCV2-infected DCs in vitro had little change [28], but PCV2 infection in vivo reduced the antigen-presentation capability of MoDCs [29]. In this study, the double-positivity rates of CD80/86 and MHC-II in PCV2-infected MoDCs also showed little changes. Contrary to the results for the single culture group, the double-positive rates for MoDCs in the endothelial IL-8 upregulation groups were significantly lower than those in the PIEC-DCs, and the double-positive rates in the endothelial IL-8 downregulation groups were significantly higher than those in the PIEC-DC group (Fig. 1C and D). Moreover, the endocytosis of MoDCs in the co-culture groups was significantly higher than that in the single culture group, and the endocytosis in the endothelial IL-8 upregulation groups was stronger (Fig. 2). Normally, the maturation rate and endocytosis of IL-8^si^-PIEC-DC group would be restored to the levels in the single culture group, but we cannot guarantee that the gene silencing efficiency was 100%. In order to eliminate the possible impact of other factors, we established the Ab-IL-8-PIEC-DC group, and found that the results of the two groups were similar. Thus, all of these findings indicated that endothelial IL-8 could inhibit the maturation of DCs, which was strengthened by PCV2.

DCs show the characteristics of transitional maturation [30]. Once the exogenous antigens invade the body, DCs adhere to endothelial cells and transendothelially migrate to peripheral tissues to capture antigens under the influence of locally secreted cytokines and chemical factors and finally mature. Endothelial IL-8 can inhibit the adhesion of leukocytes to endothelial cells [31, 32] and transendothelial migration of neutrophils [33]. The present study revealed that the adhesion and migration rate of MoDCs were negatively correlated with upregulation of endothelial IL-8 induced by PCV2 infection (Fig. 3-5). In the with-induction co-culture groups, the migration rates of MoDCs were lower than those in the after-induction co-culture groups. This may be due to the difference of the co-culture duration or the samples batch. Nevertheless, it can be inferred from the trend of change that PCV2-induced endothelial IL-8 can influence the maturity of MoDCs by affecting their adhesion and migration.

DCs that uptake virus successfully migrate into lymphoid organs and perform endogenous antigen presentation. LMP7 and UBP play vital roles in promoting antigen peptide production. A suitable MHC-I binding peptide can be obtained by LMP7 up-regulating proteasome and hydrolyzing peptide bond [34, 35]. LMP7 knockout mice show a significant decrease in MHC-I antigen-presentation function [36]. UBP can stop the proteasome from degrading target protein and control the synthesis of antigen peptide [37, 38]. Calnexin can promote the binding of the heavy chain and light chain of MHC-I and protect the heavy chain from degradation. Calnexin is replaced by calreticulin after MHC-I heterodimerization. The absence of calreticulin directly leads to the failure of MHC-I to load antigenic peptides and reduce the stability of MHC-I [39, 40]. In Fig. 6, the significantly lower expression rates of LMP7 and MHC-I in the PIEC-DC group compared to those in the single culture groups and in the endothelial IL-8 upregulation groups compared to those in the PIEC-DC group and the significantly higher expression rates in the endothelial IL-8 downregulation groups compared to that in the PIEC-DC group imply that the endothelial IL-8 induced by PCV2 inhibits the endogenous antigen-presentation function of MoDCs, which may be related to the inhibition of MoDC maturation.

Only terminally differentiated or mature DCs can activate prime T-cells, while immature DCs specialize in capturing and processing antigens [16]. The results of T-cell proliferation test (Fig. 7) showed that the SIs of after-induction co-cultures were > 1, the SIs of the co-culture groups were significantly downregulated, and those of the endothelial IL-8 upregulation groups were significantly lower than that of the PIEC-DC group, while those of the endothelial IL-8 downregulation groups were significantly higher than that of the PIEC-DC group. These findings are consistent with the maturity rates of MoDCs. It is confusing that SIs of with-induction co-cultures were < 1. We speculated that it could be related to the prolonged co-culture of MoDCs and PIECs or secretion of some apoptotic factors. However, the trend of SI change between the two induced co-cultures was similar.

## Conclusions

In summary, PCV2-induced endothelial IL-8 could affect MoDC maturation and antigen-presenting function in vitro. Whether endothelial IL-8 has the same biological function in vivo and its regulation mechanism warrants further investigation.

## Data Availability

The datasets generated and analyzed during the current study are not publicly available but are available from the corresponding author on reasonable request.

## Abbreviations

APCs: antigen-presenting cells
CMFDA: chloromethyl fluorescein diacetate
DHE: dihydroethidium
FBS: fetal bovine serum
FITC: fluorescein isothiocyanate isomer I
FQ RT-PCR: fluorescent quantitative reverse transcription PCR
LMP7: low molecular weight polypeptide
MCP-1: monocyte chemotactic protein-1
MHC: major histocompatibility complex
PK-15: Porcine kidney cell line 15
UBP: ubiquitin-specific processing protease
